# Image quality and radiologists’ subjective acceptance using model-based iterative and deep learning reconstructions as adjuncts to ultrahigh-resolution CT in low-dose contrast-enhanced abdominopelvic CT: phantom and clinical pilot studies

**DOI:** 10.1007/s00261-021-03373-5

**Published:** 2021-12-16

**Authors:** Makiko Nishikawa, Haruhiko Machida, Yuta Shimizu, Toshiya Kariyasu, Hiroyuki Morisaka, Takuya Adachi, Takehiro Nakai, Kosuke Sakaguchi, Shun Saito, Saki Matsumoto, Masamichi Koyanagi, Kenichi Yokoyama

**Affiliations:** 1 Department of Radiology, Faculty of Medicine, Kyorin University, 6-20-2 Shinkawa, Mitaka-shi, Tokyo, 181-8611 Japan; 2 Department of Radiology, Tokyo Women’s Medical University Adachi Medical Center, 4-33-1 Kohoku, Adachi-ku, Tokyo, 123-8558 Japan; 3 Department of Radiology, Kyorin University Hospital, 6-20-2 Shinkawa, Mitaka-shi, Tokyo, 181-8611 Japan; 4 Department of Radiology, University of Yamanashi, 1110 Shimokato, Chuo-shi, Yamanashi, 409-3898 Japan

**Keywords:** Abdominopelvic CT, Deep learning reconstruction, Iterative reconstruction, Radiation dose reduction, Ultrahigh-resolution CT

## Abstract

**Purpose:**

In contrast-enhanced abdominopelvic CT (CE-APCT) for oncologic follow-up, ultrahigh-resolution CT (UHRCT) may improve depiction of fine lesions and low-dose scans are desirable for minimizing the potential adverse effects by ionizing radiation. We compared image quality and radiologists’ acceptance of model-based iterative (MBIR) and deep learning (DLR) reconstructions of low-dose CE-APCT by UHRCT.

**Methods:**

Using our high-resolution (matrix size: 1024) and low-dose (tube voltage 100 kV; noise index: 20–40 HU) protocol, we scanned phantoms to compare the modulation transfer function and noise power spectrum between MBIR and DLR and assessed findings in 36 consecutive patients who underwent CE-APCT (noise index: 35 HU; mean CTDI_vol_: 4.2 ± 1.6 mGy) by UHRCT. We used paired t-test to compare objective noise and contrast-to-noise ratio (CNR) and Wilcoxon signed-rank test to compare radiologists’ subjective acceptance regarding noise, image texture and appearance, and diagnostic confidence between MBIR and DLR using our routine protocol (matrix size: 512; tube voltage: 120 kV; noise index: 15 HU) for reference.

**Results:**

Phantom studies demonstrated higher spatial resolution and lower low-frequency noise by DLR than MBIR at equal doses. Clinical studies indicated significantly worse objective noise, CNR, and subjective noise by DLR than MBIR, but other subjective characteristics were better (*P* < 0.001 for all). Compared with the routine protocol, subjective noise was similar or better by DLR, and other subjective characteristics were similar or worse by MBIR.

**Conclusion:**

Image quality, except regarding noise characteristics, and acceptance by radiologists were better by DLR than MBIR in low-dose CE-APCT by UHRCT.

**Graphical abstract:**

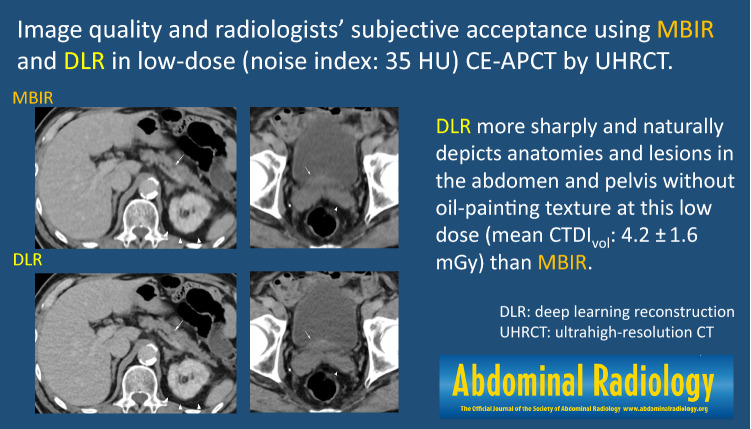

## Introduction

Since March 2017, our institution has employed an ultrahigh-resolution computed tomography (UHRCT) scanner to improve the in- and through-plane spatial resolution of CT images. The clinical utility of UHRCT has been shown in CT examinations of the temporal bone, chest, and Adamkiewicz artery and virtual bronchoscopy and coronary angiography [1–5]. However, the greater image noise associated with this method may limit its usefulness in CT that requires lower contrast resolution, such as abdominopelvic CT (APCT) for oncologic follow-up, where UHRCT may improve diagnosis of fine recurrent, disseminated, and metastatic lesions.

To overcome these potential limitations, deep learning (DLR) and model-based iterative (MBIR) reconstruction techniques have become clinically available for use in combination with UHRCT [6]. MBIR is reported to improve image quality with respect to noise characteristics, spatial resolution, artifacts, and low-contrast detectability. However, radiologists have been reluctant to adopt this modality because it produces a coarse texture associated with low-frequency noise, described as an “oil painting” or “plastic-like” appearance, compared to results obtained using hybrid iterative reconstruction (HIR), which is widely applied in clinical settings [6–8]. At a routine radiation dose, the quality of abdominal UHRCT images may be better using DLR than either MBIR or HIR [6]. On the other hand, contrast-enhanced APCT (CE-APCT) for oncologic follow-up requires a relatively large amount of contrast material (CM) and a high radiation dose [9–11]. Minimizing CM dose may be desirable for oncology patients because they tend to have multiple risk factors for kidney injury [12–14] and reduction of radiation dose is critical to minimize the potential adverse effects by ionizing radiation because repeated CT examinations usually need to be performed [15]. In particular, lowering tube voltage may enable reasonable low-radiation-dose (LD) UHRCT scans aided by DLR while preserving contrast enhancement even with reduction of CM dose. We believe, though, that the quality and acceptance by radiologists of LD CE-APCT images by UHRCT using DLR as well as MBIR has not been assessed yet.

We therefore undertook phantom and clinical pilot studies by UHRCT to compare findings between MBIR and DLR in CE-APCT for oncologic follow-up obtained utilizing a high-resolution and LD (HR & LD) protocol using our routine protocol as reference. We evaluated the image quality and radiologists’ acceptance of MBIR and DLR and attempted to determine the appropriate HR & LD protocol that would yield the least radiation exposure.

## Materials and methods

In this study, we mainly aimed to (1) determine the HR & LD protocol with MBIR and/or DLR to achieve the lowest radiation dose and the similar low-frequency noise to that using the routine protocol in the phantom study and (2) assess validity of this HR & LD protocol based on image quality and radiologists’ acceptance using the routine protocol as reference in the clinical pilot study.

Our institutional review board approved this clinical study, and we obtained written informed consent from all patients.

### Phantom study

#### Phantoms

**Fig. 1:**
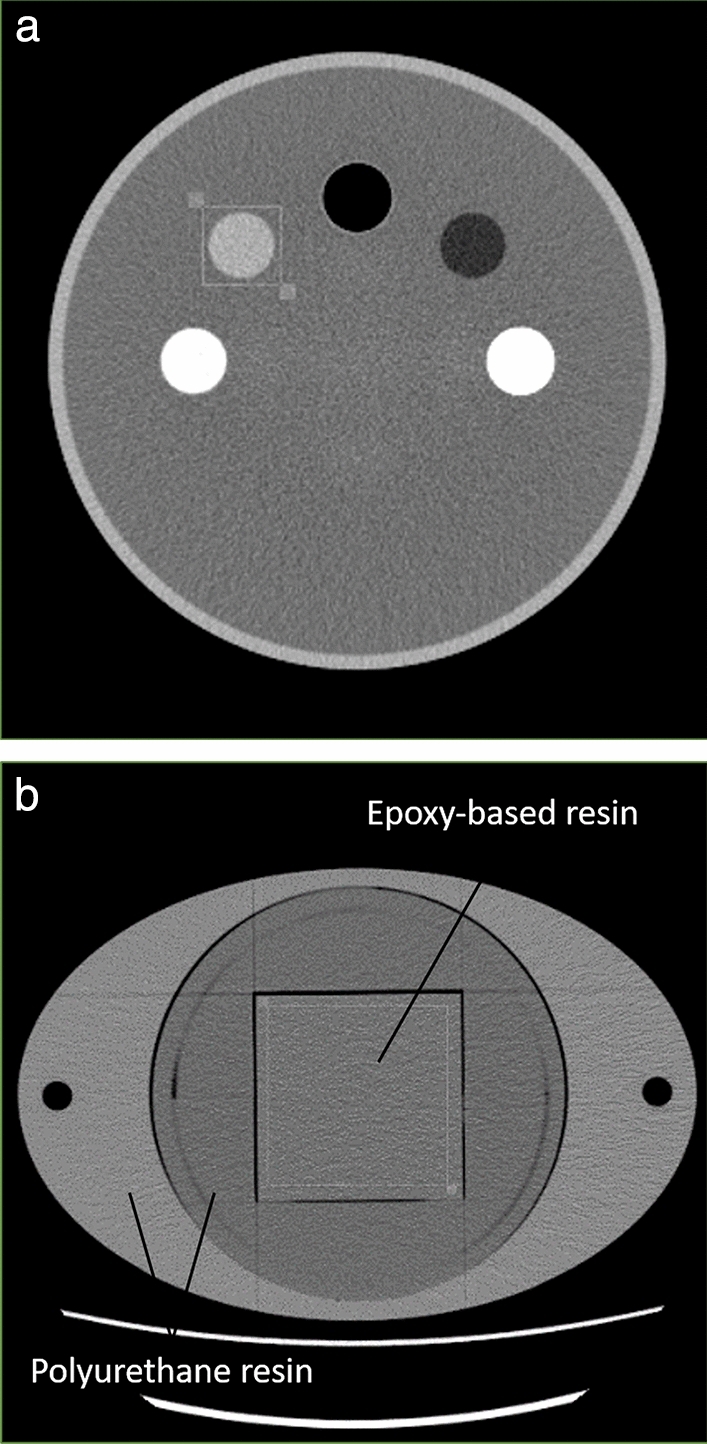
Axial CT images of the 2 phantoms used to assess **a** the task-based modulation transfer function (TTF), which included cylinder inserts of various materials that offered different image contrasts (left to right: Delrin, 340 HU; acrylic, 120 HU; air, -1000 HU; polypropylene, -105 HU; and Teflon, 940 HU), and **b** the noise power spectrum (NPS), made primarily of epoxy-based and polyurethane resin

We assessed spatial resolution by the task-based modulation transfer function (TTF) using a quality assurance phantom (TOS phantom; Canon Medical Systems, Tochigi, Japan) that included inserts of various materials to provide different levels of image contrast (air, -1000 HU; polypropylene, -105 HU; water, 0 HU; acrylic, 120 HU; Delrin, 340 HU; and Teflon, 940 HU). We focused on the acrylic insert of the lowest positive contrast, almost equivalent to the contrast between soft tissue and fat attenuations, for oncologic follow-up by CE-APCT. To assess image noise characteristics by the noise power spectrum (NPS), we utilized an original abdomen phantom comprising an elliptical cylinder (33-cm longest diameter, 22-cm shortest diameter) made of epoxy-based and polyurethane resin (Kyoto Kagaku, Kyoto, Japan) (Fig. 1) [16].

#### CT image acquisition and reconstruction

We performed helical scanning of the 2 phantoms with a UHRCT scanner (Aquilion Precision, Canon Medical Systems) using automatic exposure control (AEC) and parameters for our routine and HR & LD protocols, which are summarized in Table 1. Specifically, we used 5 dose settings at AEC noise indices of 20, 25, 30, 35, and 40 HU for the HR & LD protocol at 100 kV. To assess radiation exposure, we reviewed the volume CT dose index (CTDI_vol_) for each protocol recorded as a dose report.

**Table 1:** Parameters for CT scanning and reconstruction

Protocol	Routine	High-resolution and low-radiation-dose
Scan parameters		
Tube voltage	120 kV	100 kV
Tube current^a^	15 HU	20, 25, 30, 35, and 40 HU
Rotation time	0.5 s	
Field of view	50 cm (phantom study); 32–50 cm (clinical study)	
Number of detector channels	896	1792
Slice collimation	0.5 mm × 80	
Pitch factor	0.812	
Focus size	0.6 mm × 1.3 mm	
Reconstruction parameters		
Algorithm	HIR (AIDR 3D Standard)	MBIR (FIRST Body Standard); DLR (AiCE Body Standard)
Kernel	FC03	Not applicable
Matrix size	512	1024
Slice thickness	0.5 mm	
Field of view	35 cm (phantom study); 32–45 cm (clinical study)	

^a^The noise index was set as the standard deviation of the CT number for 5-mm thickness and the use of a standard kernel with filtered back projection with automatic exposure control. The single noise index of 35 HU was used in the high-resolution and low-radiation-dose protocol for the clinical pilot study

*AiCE* advanced intelligent clear-IQ engine, *AIDR* adaptive iterative dose reconstruction, *DLR* deep learning reconstruction, *FIRST* forward-projected model-based iterative reconstruction, *HIR* hybrid iterative reconstruction, *MBIR* model-based iterative reconstruction

We reconstructed the phantom images using a standard kernel (FC03) and an HIR algorithm (Adaptive Iterative Dose Reconstruction [AIDR] 3D Standard, Canon Medical Systems) for those acquired with the routine protocol and using an MBIR algorithm (Forward-projected model-based Iterative ReconSTruction [FIRST] Body Standard, Canon Medical Systems) and a DLR algorithm (Advanced intelligent clear-IQ engine [AiCE] Body Standard, Canon Medical Systems) for those acquired with the HR & LD protocol. Table 1 summarizes other reconstruction parameters.

**Fig. 2:**
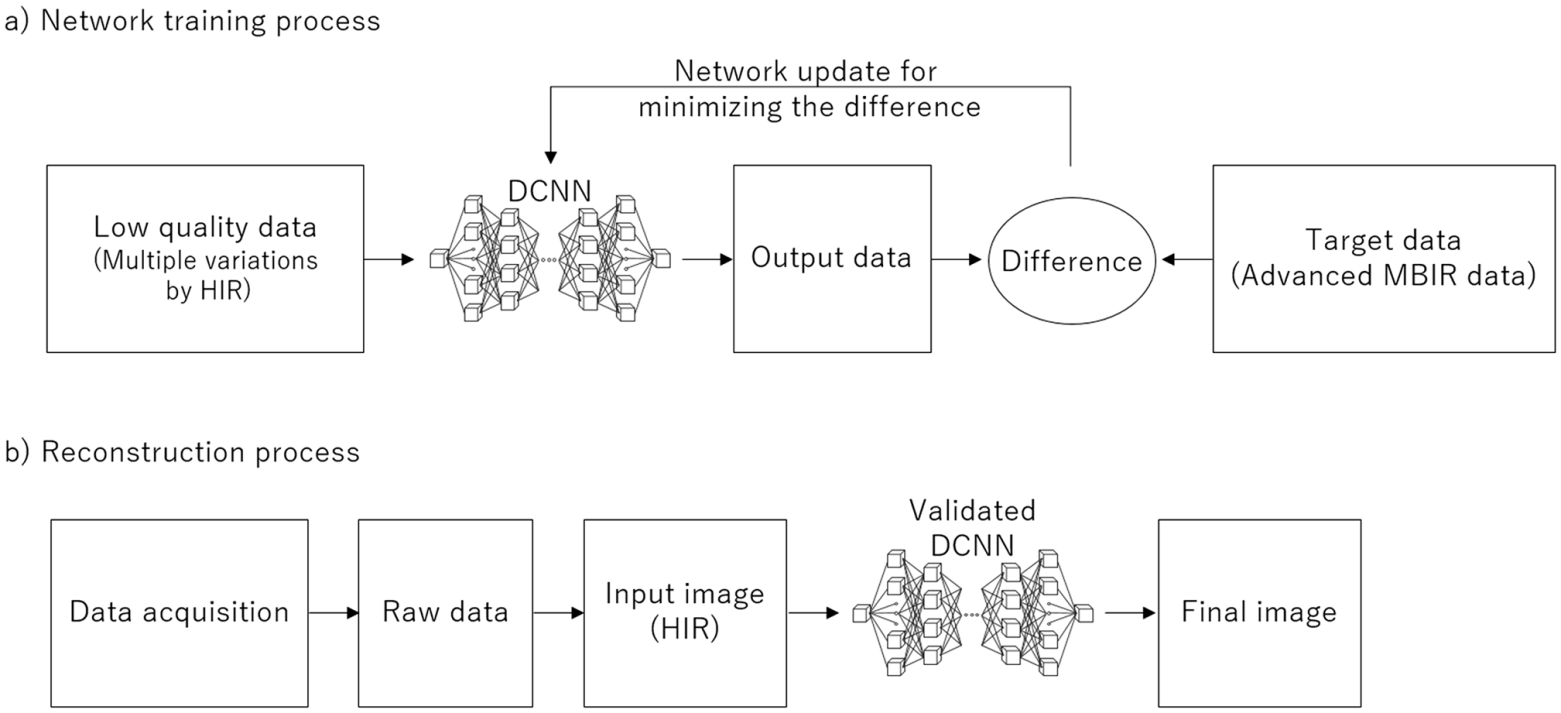
Flowcharts of the training and reconstruction process in deep learning reconstruction (DLR). **a** In the training process, given standard-dose images by hybrid iterative reconstruction (HIR) as low-quality input data and high-dose images by advanced model-based iterative reconstruction (MBIR) with much more iterations than MBIR as targeting high-quality data as training pairs, the deep convolutional neural network (DCNN) is updated to minimize the difference between DCNN output and the target for future reconstructions. This process has been performed in advance as a black box by the manufacturer. **b** In the reconstruction process, the DCNN is validated for clinical image processing to generate final high-quality images from input images by HIR

For the DLR, as shown in Fig. 2, standard-dose images by HIR as low-quality input data and high-dose images by advanced MBIR with much more iterations than MBIR as targeting high-quality data were used as training pairs, and in the training process, statistical features that differentiate signals from the noise and artifacts could be “learned” and then “updated” in the deep convolutional neural network for use in future reconstructions [6, 17]. This training process had been performed in advance as a black box by the manufacturer. Because these ideal MBIR images were used to train the network, DLR yielded comparable or superior image quality to that of MBIR in a shorter processing time than that of MBIR [6].

#### Image quality assessment

We analyzed each set of phantom images using the appropriate software (CT measure version 0.98, http://www.jsct-tech.org/; Excel 2016, Microsoft). On an axial image of the acrylic insert in the quality assurance phantom, we radially acquired and averaged profile curves crossing the circular edge to obtain its edge-spread function. TTF was calculated by Fourier transformation using line-spread function obtained by differentiating the edge-spread function to assess the intermediate-contrast in-plane spatial resolution with non-linear algorithms, such as HIR, MBIR, and DLR, at various noise levels. Our method to determine NPS in the epoxy-based resin part is described elsewhere [8, 16]. Using routine images reconstructed by HIR for reference, we then compared TTF and NPS between the HR & LD images with the 5 dose settings at noise indices of 20, 25, 30, 35, and 40 HU reconstructed by MBIR and DLR. Ultimately, we determined the noise index setting for the AEC in the HR & LD protocol by MBIR and/or DLR to minimize the radiation dose while preserving or lessening noise at lower frequencies compared with the noise obtained using the routine protocol.

### Clinical pilot study

#### Subjects

From March 11 through March 29, 2019, we prospectively enrolled 41 consecutive adult patients with mild to moderate renal impairment (i.e., estimated glomerular filtration rate: 30 to 59 mL/min/1.73m^2^) from whom we obtained written informed consent and who underwent CE-APCT using an HR & LD protocol with the UHRCT scanner for oncologic follow-up. Exclusion criteria were: inadequate CT image acquisition and history of surgical operation of the liver and/or intrapelvic organs, which precluded our image quality assessment described below. Actually, 5 patients were excluded due to insufficient scan coverage (*n* = 3) and history of total hysterectomy (*n* = 2). Thus, we finally included 36 consecutive patients (24 men, 12 women; mean age, 75 ± 9 years; range, 48 to 93 years; mean body weight [BW], 57.1 ± 11.7 kg; range, 39 to 86 kg; mean body mass index [BMI], 22.5 ± 3.5 kg/m^2^; range, 15 to 29 kg/m^2^) in the present study. Using the routine protocol as reference, we compared the quality of the CT images reconstructed by MBIR and DLR. In the HR & LD protocol, we set the noise index at 35 HU based on the results of the aforementioned phantom study, as described in the “Results” section, and reduced the iodine load by 40% of that with our routine protocol.

#### CT image acquisition and reconstruction

Patients underwent helical acquisition of CE-APCT with the UHRCT scanner using parameters summarized in Table 1. All patients received non-ionic iodinated CM (Iopamiron 300; Bayer HealthCare, Osaka, Japan) at a concentration of 300 mgI/mL. A total dose of 312 mgI/kg of BW was administered over 45 s via the right antecubital vein using a 22-gauge plastic intravenous catheter with a power injector (Dual Shot-type GX 7; Nemoto Kyorindo, Tokyo, Japan), and scanning began at 120 s following the start of CM administration. To assess radiation exposure, we reviewed the CTDI_vol_ and dose-length product (DLP) recorded as a dose report and then calculated the estimated effective dose as the DLP multiplied by a k factor for the abdomen and pelvis of 0.015 mSv mGy^−1^ cm^−1^ [18] for each patient. Thus, we calculated the mean CTDI_vol_, DLP, and estimated effective dose for the HR & LD protocol. As in the phantom study, we used both the MBIR and DLR algorithms to reconstruct the CE-APCT images acquired for each patient (Table 1).

#### Quantitative assessment of image quality

On the CT images reconstructed by both MBIR and DLR and displayed on a commercially available workstation (Ziostation Version 2.4; Ziosoft, Inc., Tokyo, Japan), 3 radiology technologists, by consensus, employed a copy-and-paste function to place 3 circular regions of interest (ROIs) in the hepatic parenchyma, carefully avoiding large vessels and any areas of focal changes in attenuation, and prominent artifacts. In a similar manner, they placed a circular ROI in the upper abdominal subcutaneous fat at the same level, in the major psoas muscle at the level of the aortic bifurcation, avoiding any macroscopic fat infiltration, in the urinary bladder and the prostate (for men) or uterus (for women), avoiding any areas of focal change in attenuation and prominent artifacts, and in the lower abdominal subcutaneous fat at the same level. Thus, they measured the CT number and its standard deviation (SD) value within these anatomies in each patient. We calculated the mean SD value in all patients as objective noise in the hepatic parenchyma, upper abdominal subcutaneous fat, major psoas muscle, urinary bladder, and lower abdominal subcutaneous fat. We also calculated the contrast-to-noise ratio (CNR) of the liver and pelvis using the following equations: CNR of the liver = (mean CT number of the hepatic parenchyma – CT number of the major psoas muscle)/noise in the upper abdominal subcutaneous fat, and CNR of the pelvis = (CT number of the prostate or uterus – CT number in the urinary bladder) / noise in the lower abdominal subcutaneous fat.

#### Qualitative assessment of image quality

On the workstation, 2 independent board-certified radiologists with 10 and 11 years’ clinical experience who were blinded to patient demographics and CT parameters used a 5-point scale to grade the quality of CE-APCT images reconstructed by both MBIR and DLR. Five points represented much better quality compared with the reference; 4 points, better quality; 3 points, comparable quality; 2 points, worse quality; and one point, much worse quality. Referencing routine CE-APCT images of other 101 consecutive adult patients (52 men, 49 women; mean age, 65 ± 17 years; range, 29 to 92 years; mean BW, 57.3 ± 14.4 kg; range, 32 to 117 kg; mean BMI, 21.9 ± 4.5 kg/m^2^; range, 12 to 36 kg/m^2^) with normal renal function (i.e., estimated glomerular filtration rate: ≥ 60 mL/min/1.73m^2^) imaged by UHRCT using the routine scan and reconstruction protocol (Table 1) and our routine iodine load (520 mgI/kg of BW) from January 1 through March 1, 2019, the reviewers considered the general acceptability of the image with regard to overall diagnostic confidence and both image appearance and image texture in the liver and intrapelvic organs (prostate or uterus and urinary bladder) as well as image noise, as described by Laurent and colleagues [7]. The HR & LD images reconstructed by both MBIR and DLR were presented in random order on a preset soft tissue window (window width, 370 HU; window level, 40 HU).

#### Statistical analysis

Results were expressed as mean ± SD for continuous variables. Statistical analysis was performed using commercially available statistical software (SPSS for Windows, Version 23.0, IBM SPSS, Armonk, NY). Objective noise and CNR were compared between MBIR and DLR using paired t-test, and subjective image quality grades were compared using Wilcoxon signed-rank test. BW and BMI were compared between the study and reference patient groups using unpaired t-test. A *P* value below 0.05 was considered to indicate significant difference. Inter-reviewer agreement was estimated using weighted kappa statistics.

## Results

### Phantom study

**Fig. 3:**
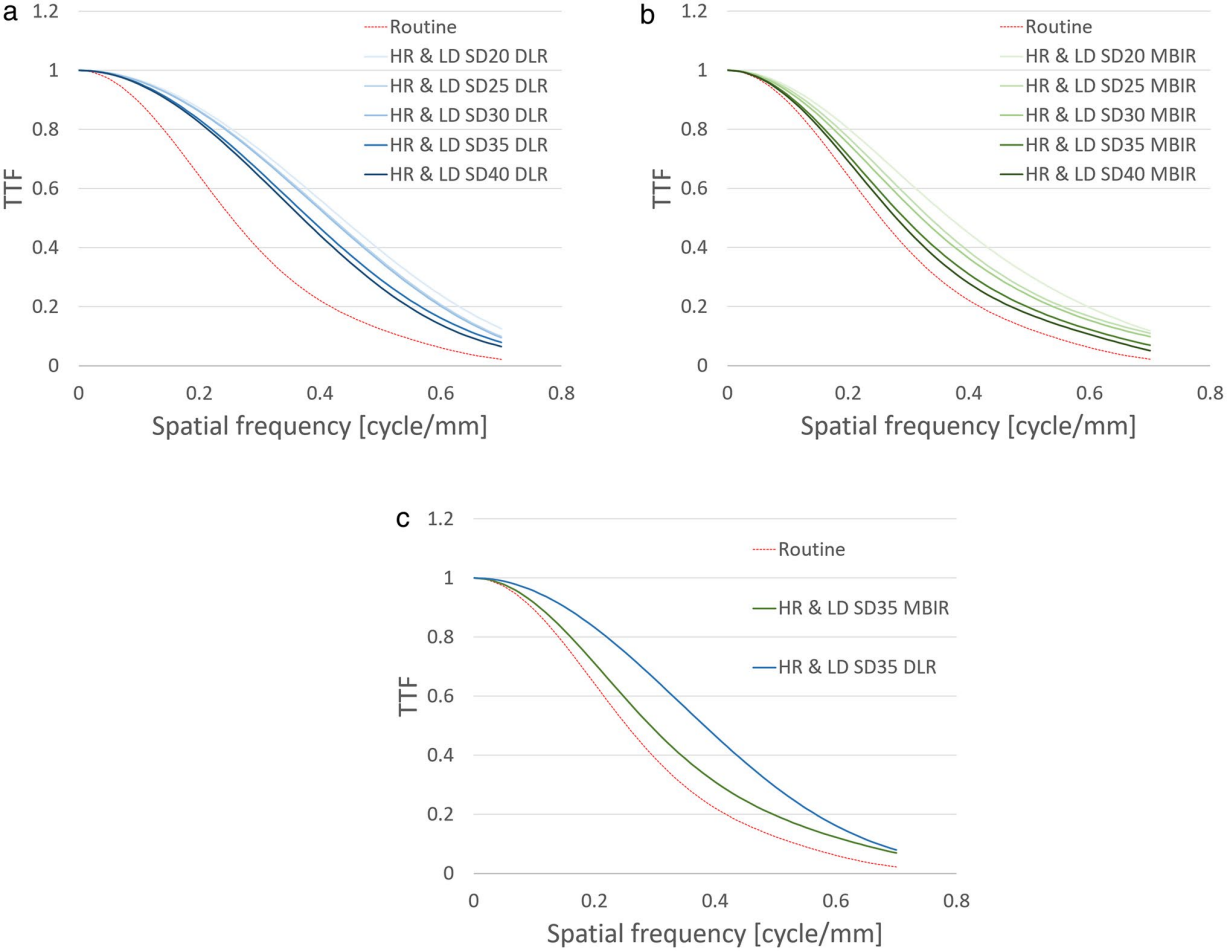
Task-based modulation transfer function (TTF) curves for deep learning reconstruction (DLR) (**a** and **c**: blue-tone curves) and model-based iterative reconstruction (MBIR) (**b** and **c**: green-tone curves) at 5 different dose levels (**a** and **b**), particularly including standard deviation (SD) of 35 HU (**c**), using the high-resolution and low-radiation-dose (HR & LD) protocol with those for hybrid iterative reconstruction (HIR) using our routine protocol (**a**–**c**: red dotted curves). Spatial resolution is higher for both DLR and MBIR using the HR & LD protocol than that for HIR using the routine protocol. Note the higher spatial resolution for DLR than MBIR at the same radiation dose

**Fig. 4:**
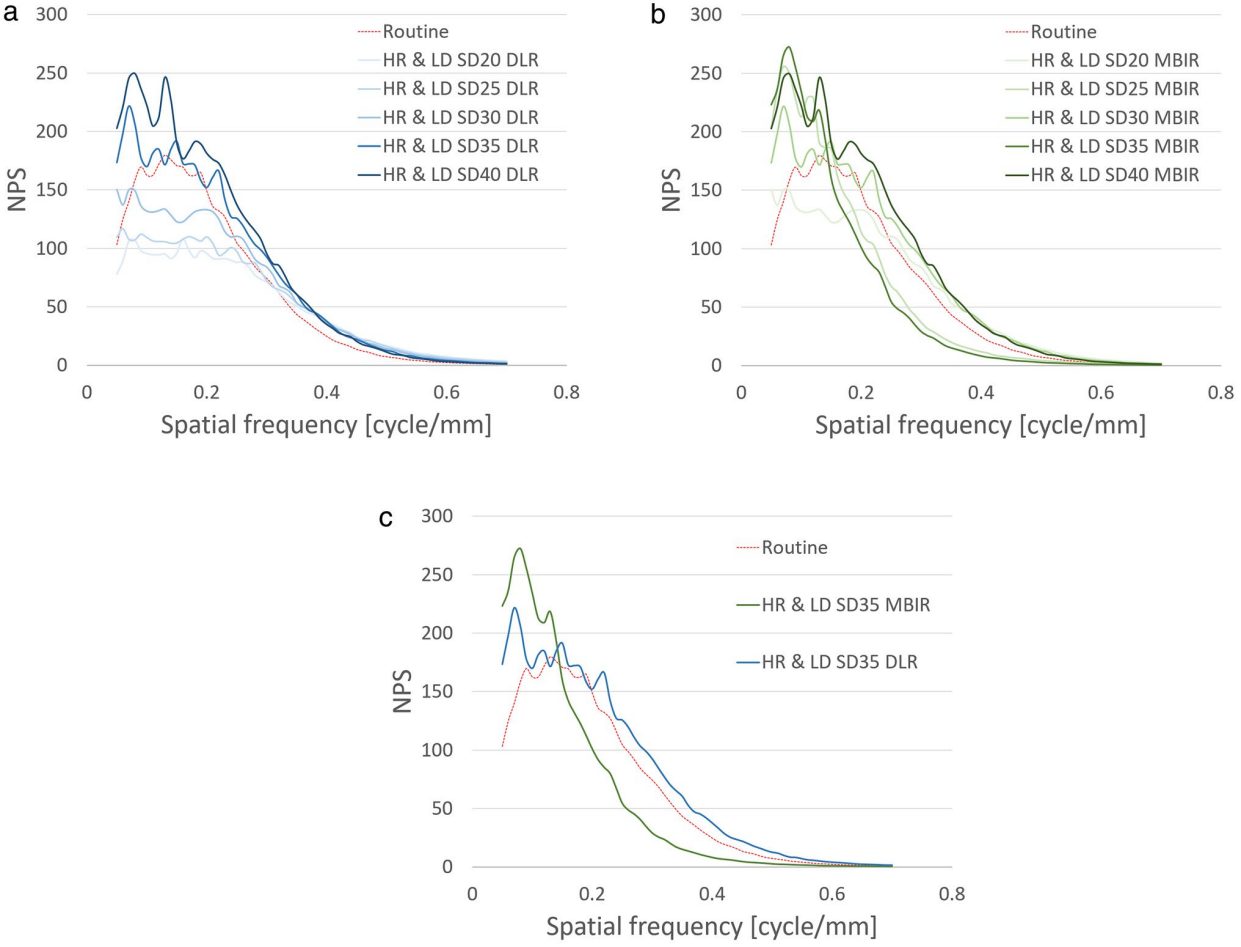
Noise power spectrum (NPS) curves for deep learning reconstruction (DLR) (**a** and **c**: blue-tone curves) and model-based iterative reconstruction (MBIR) (**b** and **c**: green-tone curves) at 5 different dose levels (**a** and **b**), particularly including standard deviation (SD) of 35 HU (**c**), using the high-resolution and low-radiation-dose (HR & LD) protocol with those for hybrid iterative reconstruction (HIR) using our routine protocol (**a**–**c**: red dotted curves). Low-frequency noise is less for DLR than for MBIR at the same radiation dose. Note that the noise for HIR using the routine protocol is comparable to that for DLR at an SD of 35 HU and less than that for MBIR at an SD of 35 HU (**c**)

In the phantom study, the CTDI_vol_ was 8.7 mGy using the routine protocol and 11.3 mGy at a noise index of 20 HU; 9.7 mGy, 25 HU; 7.2 mGy, 30 HU; 5.5 mGy, 35 HU; and 4.4 mGy, 40 HU using the HR & LD protocol. As shown in Fig. 3, the phantom study revealed a higher TTF with the HR & LD protocol than with the routine protocol, and TTF was higher by DLR than MBIR at the same dose with the HR & LD protocol. This tendency was more prominent at lower doses. In addition, the HR & LD protocol yielded less low-frequency noise but greater high-frequency and overall noise (i.e., SD value; calculated by area under the NPS curve) by DLR than by MBIR at the same dose (Fig. 4). In particular, low-frequency noise was less at a noise index of 20–30 HU, comparable at 35 HU, and higher at 40 HU by DLR, but it was higher at 35–40 HU by MBIR compared with the routine protocol. We thus determined to use DLR at a noise index of 35 HU as the HR & LD protocol for our clinical pilot study to minimize radiation exposure and achieve similar image texture and greater sharpness compared to those with the routine protocol. Actually, the CTDI_vol_ at an index of 35 HU (i.e., 5.5 mGy) with the HR & LD protocol was lower than that using the routine protocol (i.e., 8.7 mGy); TTF increased from that using the routine protocol to that at an index of 35 HU with MBIR to that at an index of 35 HU by DLR (Fig. 3c); low-frequency noise at an index of 35 HU by DLR was comparable to that using the routine protocol and less than that at an index of 35 HU by MBIR (Fig. 4c). Nevertheless, overall noise increased from that at an index of 35 HU with MBIR (6.9 HU) to that using the routine protocol (7.2 HU) to that at an index of 35 HU by DLR (8.4 HU).

### Clinical pilot study

**Fig. 5:**
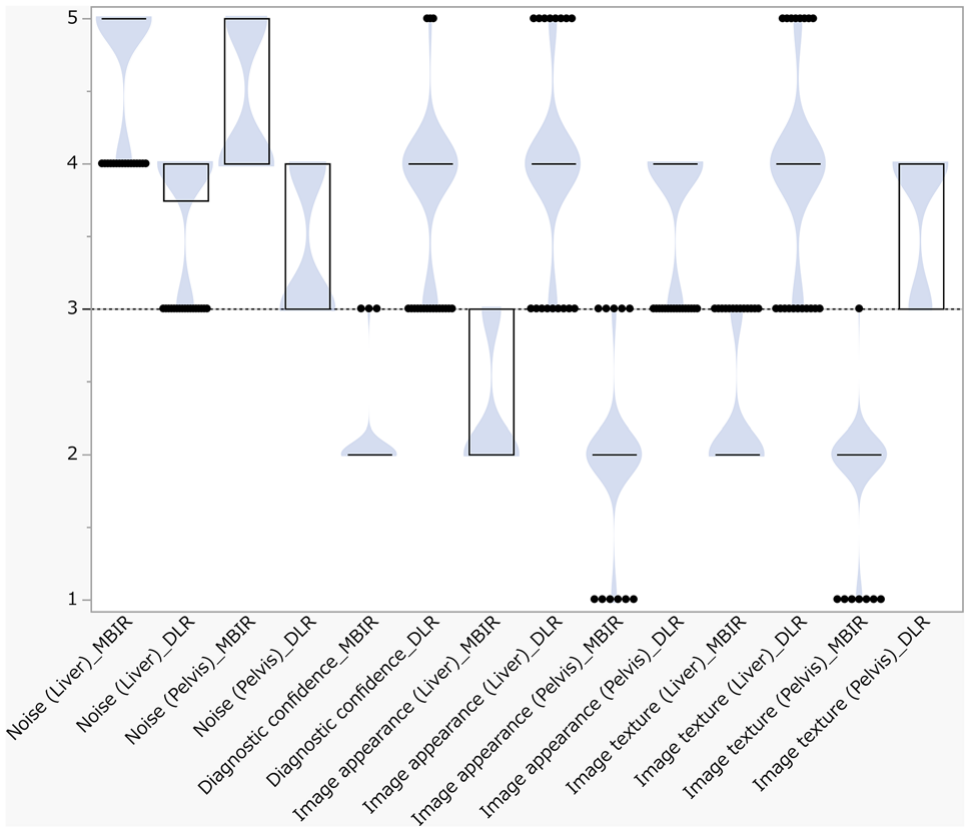
Violin plots with box-and-whisker plots representing the subjective image quality scores by model-based iterative reconstruction (MBIR) than by deep learning reconstruction (DLR) in the clinical pilot study. Five points represent much better quality compared with the routine protocol; 4 points, better quality; 3 points, comparable quality; 2 points, worse quality; and one point, much worse quality. The dashed line represents 3 points. Use of the high-resolution and low-radiation-dose protocol at a noise index of 35 HU yields significantly less subjective noise in both the liver and pelvis, but all subjective image qualities except the noise are significantly worse by MBIR than by DLR (*P* < 0.001 for all). Note that all the scores are 3 to 5 by DLR, representing non-inferior diagnostic efficacy by DLR compared to the routine protocol

**Fig. 6:**
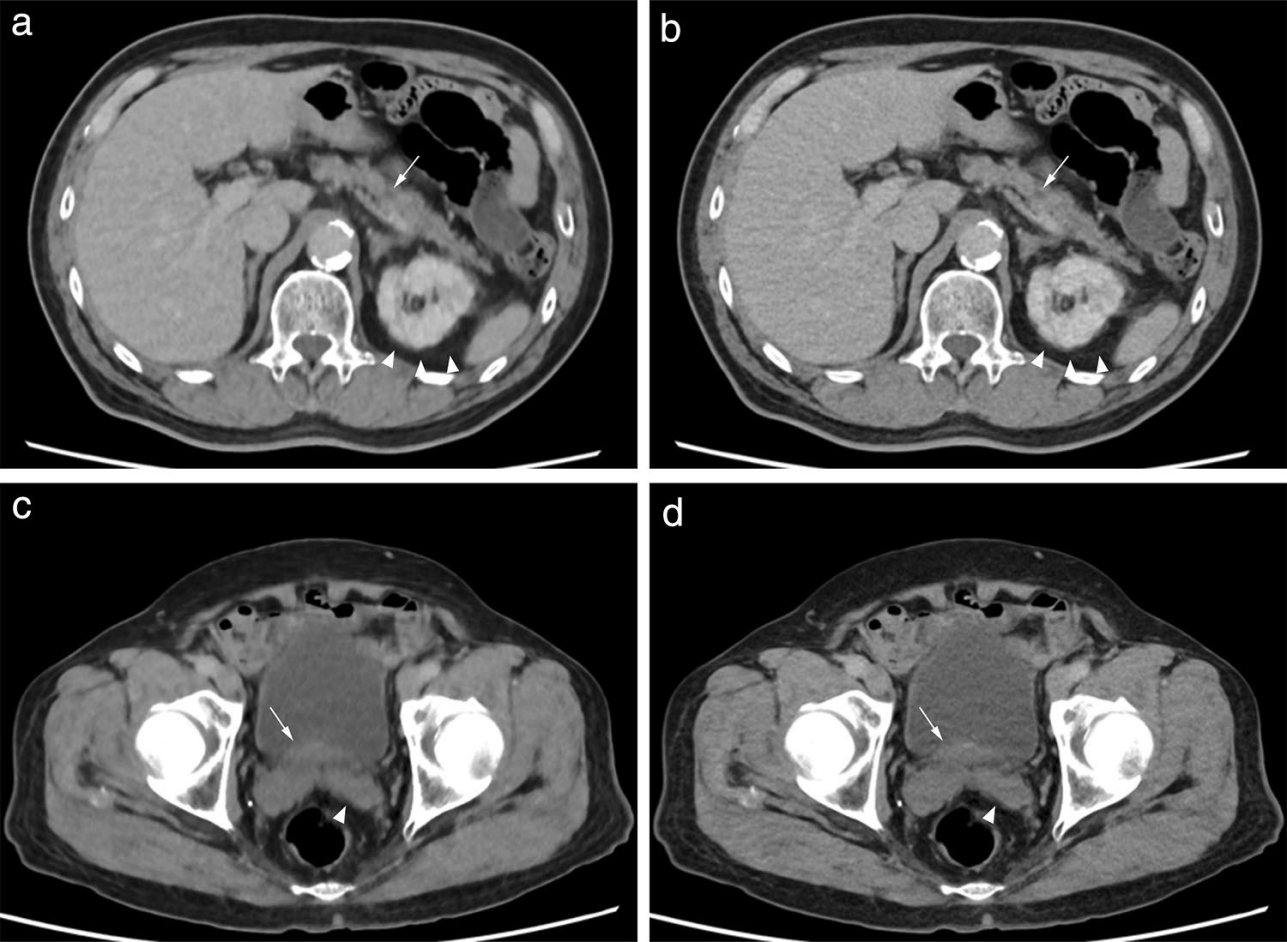
Contrast-enhanced ultrahigh-resolution CT (UHRCT) axial images of the abdomen (**a** and **b**) of a 71-year-old man (156 cm, 60 kg, body mass index [BMI]: 24.7 kg/m^2^) and the pelvis (**c** and **d**) of an 86-year-old man (152 cm, 56 kg, BMI: 24.2 kg/m^2^) acquired with the high-resolution and low-radiation-dose protocol (tube voltage, 100 kV; standard deviation [SD], 35 HU; CT dose index volume, 4.7 mGy for the first subject and 4.0 mGy, for the second) and reconstructed by model-based iterative reconstruction (MBIR) (**a** and **c**) and deep learning reconstruction (DLR) (**b** and **d**). Despite its greater subjective noise, DLR more sharply and naturally depicts anatomies in the abdomen and pelvis than MBIR without the characteristic oil painting texture of MBIR. Specifically, the delineation of the cystic lesion in the pancreatic body (arrows) and the Gerota’s fasciae (arrowheads) is more conspicuous by DLR (**b**) than by MBIR (**a**); the boundaries between the prostate (arrows) and the adjacent urine in the urinary bladder and between the seminal vesicles (arrowheads) and the surrounding fat tissue appear clearer by DLR (**d**) than by MBIR (**c**)

In the clinical pilot study, use of the HR & LD protocol at a noise index of 35 HU yielded significantly less objective and subjective noise and significantly greater CNR in all anatomies, but all subjective image qualities except subjective noise were significantly worse by MBIR than by DLR (*P* < 0.001 for all, Table 2 and Figs. 5 and 6). Both reviewers graded subjective noise as 4 or 5 by MBIR and 3 to 5 by DLR in all patients, and they scored all other subjective image quality from one to 3 by MBIR and 3 to 5 by DLR. Inter-reviewer agreement was excellent (κ = 0.87). Both BW and BMI were comparable between the study and reference patient groups (*P* = 0.892 and 0.512, respectively). With the HR & LD protocol, the mean CTDI_vol_ was 4.2 ± 1.6 mGy, the DLP, 243.2 ± 106.0 mGy cm, and the estimated effective dose, 3.6 ± 1.6 mSv.

**Table 2:** Objective noise, contrast-to-noise ratio, and subjective image quality

	MBIR	DLR	*P* value
Objective noise (HU)			
Liver	6.4 ± 0.1	9.5 ± 0.1	< 0.001
Upper abdominal subcutaneous fat	7.0 ± 2.3	8.7 ± 1.9	< 0.001
Psoas muscle	6.4 ± 1.4	9.0 ± 1.4	< 0.001
Urinary bladder	6.4 ± 1.4	9.2 ± 1.6	< 0.001
Lower abdominal subcutaneous fat	7.4 ± 1.9	9.3 ± 1.7	< 0.001
CNR			
Liver	5.2 ± 0.0	4.2 ± 0.1	< 0.001
Pelvis	6.8 ± 0.2	5.3 ± 0.0	< 0.001
Subjective image quality			
Noise (liver)	4.8 ± 0.1	3.8 ± 0.0	< 0.001
Noise (pelvis)	4.4 ± 0.0	3.4 ± 0.0	< 0.001
Diagnostic confidence	2.0 ± 0.2	3.8 ± 0.2	< 0.001
Image appearance (liver)	2.2 ± 0.0	4.0 ± 0.0	< 0.001
Image appearance (pelvis)	2.0 ± 0.1	3.8 ± 0.1	< 0.001
Image texture (liver)	2.2 ± 0.0	4.0 ± 0.0	< 0.001
Image texture (pelvis)	2.0 ± 0.1	3.7 ± 0.1	< 0.001

*CNR* contrast-to-noise ratio, *DLR* deep learning reconstruction, *MBIR* model-based iterative reconstruction

## Discussion

The phantom study, using the HR & LD protocol, demonstrated higher spatial resolution and lower low-frequency noise by DLR than by MBIR at the same dose. Compared with findings using the routine protocol, low-frequency noise was similar by DLR but greater by MBIR at a noise index of 35 HU, whereas high-frequency and overall noise were greater by DLR and less by MBIR. Low-frequency noise was reported to produce coarse image texture described as an “oil painting” or “plastic-like” appearance, which compromised the detection of small lesions [7, 8, 19]. From the clinical pilot study, using this imaging protocol at a noise index of 35 HU, though noise characteristics were worse by DLR than by MBIR, the objective noise was less than 10 HU even by DLR, and both reviewers graded subjective noise as similar or better in all patients compared with findings using the routine protocol. An optimal noise index of 12.5 or 15.0 HU was reported to obtain diagnostically acceptable APCT images at a reasonably reduced radiation dose using conventional multidetector CT (MDCT) scanners and filtered back projection (FBP) [20]. The other image qualities‒diagnostic confidence, image appearance, and image texture‒were similar or better by DLR but similar or worse by MBIR in all patients compared to findings with the routine protocol, and they were significantly better by DLR than by MBIR. Because the patient body size was comparable between the study and reference groups, all the subjective image qualities and thus diagnostic efficacy were thought to be not inferior only by DLR (i.e., not by MBIR) using the HR & LD protocol compared with the routine protocol including the standard resolution and dose and HIR.

Reporting clinical study findings, Akagi and colleagues [6] observed that DLR improved the quality of abdominal CT images obtained by UHRCT at their routine dose (CTDI_vol_: 12.6 mGy). Generally, attenuation is greatest as the x-ray beam travels horizontally at the level of the hip joint because the pelvic bones and bilateral femoral heads block photons from reaching the x-ray detectors, resulting in photon starvation artifact as a major issue to be resolved in low-dose pelvic CT [21]. We first applied the HR & LD protocol in APCT by UHRCT clinically and successfully reduced radiation dose by two-thirds (i.e., CTDI_vol_: 4.2 mGy) compared with the study by Akagi’s group [6], achieving a value much lower than the diagnostic reference levels for low-dose APCT followed in many countries (CTDI_vol_: 13 to 18 mGy) [22, 23]. This reduced dose still permitted acquisition of adequate image quality in CE-APCT by UHRCT for oncologic follow-up, though such challenges are perceived as more easily manageable in certain high-contrast examinations, such as CT angiography, CT examination of nephroureterolithiasis, and CT colonography [24, 25]. Previous studies reduced the CTDI_vol_ to approximately 6 mGy in APCT examinations by conventional MDCT (i.e., non-UHRCT) with tube voltage reduction and/or the application of various iterative reconstruction algorithms [21, 26–35]. Park and colleagues [26] described the combined use of automated attenuation-based tube potential selection on third-generation dual-source CT with an iterative reconstruction algorithm maximized median CTDI_vol_ reduction. They were able to achieve a median CTDI_vol_ of 4.8 mGy by decreasing tube voltage to 90 kV in a patient subgroup with the smallest body physique, but their result is still higher than the mean CTDI_vol_ in the present study. In a phantom study utilizing a conventional MDCT scanner, Higaki and colleagues [8] reported less low-frequency noise and thus higher task-based detectability at various task contrast settings by DLR than MBIR at low radiation doses. The exact reason is unknown; however, DLR is robust in low-dose situations because its training includes low-quality datasets to allow the generation of high-quality images from low-quality images with the preservation of signal and spatial resolution [white paper, https://mfl.ssl.cdn.sdlmedia.com/636837173033229994OU.pdf. Accessed 24 Apr 2020]. In our study, noisier UHRCT images probably enhanced these benefits by DLR. In contrast, spatial resolution by MBIR is easily degraded in low-dose and/or low-contrast situations [36]. MBIR is associated with changes in image texture related to the distribution of signal within a narrow bandwidth of frequencies compared to that with HIR, which accounts for the coarser texture of MBIR images [7]. This texture change by MBIR might degrade subjective image qualities other than subjective noise by radiologists with a clear preference toward HIR. In addition, higher spatial resolution allows sharper delineation of various anatomies and more conspicuous depiction of potential focal lesions by DLR than MBIR. As well, MBIR usually requires higher computational power and longer processing time than those with FBP and HIR [6], and with shorter and more reasonable processing time, DLR is considered more clinically useful than MBIR [6]. Thus, the use of DLR is regarded as clinically acceptable as an adjunct to CE-APCT by UHRCT with the HR & LD protocol because it yields similar or better subjective image qualities and thus non-inferior diagnostic efficacy compared to those acquired using the routine protocol. The combination of DLR and the HR & LD protocol may be particularly beneficial for maximally reducing radiation dose and improving diagnosis of fine recurrent, disseminated, and metastatic lesions in CE-APCT by UHRCT for oncologic follow-up.

Our study was limited as follows. In the phantom study, we assessed TTF only using the single intermediate contrast, almost equivalent to image contrast between attenuations of soft tissue lesions (e.g., peritoneal disseminations, lymph node metastases) and intra- and retroperitoneal fat, instead of multiple contrasts including a low contrast for metastases within solid organs. In the clinical study, it included only a small study population at a single institution, and the smaller BW and BMI of our Japanese patients compared with those of average-sized patients in Western countries may have affected our findings. In addition, we assessed only image quality in CE-APCT but did not examine lesion delineation or diagnostic performance. Further studies to assess the clinical usefulness of our results should include examination of lesion delineation and diagnostic performance in a larger cohort at multiple institutions.

## Conclusions

In CE-APCT at a low dose (CTDI_vol_: approximately 4 mGy) by UHRCT, DLR yields better image qualities, with the exception of noise characteristics, and greater acceptance by radiologists than the use of MBIR. Particularly, lower low-frequency noise is likely to produce less coarseness of image texture and better acceptance by radiologists at the low dose by DLR than by MBIR.

## Abbreviations

AEC: Automatic exposure control
AiCE: Advanced intelligent clear-IQ engine
AIDR: Adaptive iterative dose reconstruction
APCT: Computed tomography of the abdomen and pelvis
BMI: Body mass index
BW: Body weight
CE-APCT: Contrast-enhanced APCT
CM: Contrast material
CNR: Contrast-to-noise ratio
CT: Computed tomography
CTDIvol: CT dose index volume
DLP: Dose-length product
DLR: Deep learning reconstruction
DCNN: Deep convolutional neural network
FBP: Filtered back projection
FIRST: Forward-projected model-based iterative reconstruction
HIR: Hybrid iterative reconstruction
HR & LD: High-resolution and low-radiation-dose (protocol)
LD: Low-radiation-dose
MBIR: Model-based iterative reconstruction
MDCT: Multidetector computed tomography
MTF: Modulation transfer function
NPS: Noise power spectrum
ROI: Region of interest
SD: Standard deviation
TTF: Task-based modulation transfer function
UHRCT: Ultrahigh-resolution computed tomography
